# Dynamical Regulation Analysis Identifies Molecular Mechanisms of Fuzheng-Huayu Formula against Hepatitis B-Caused Liver Cirrhosis

**DOI:** 10.1155/2015/238495

**Published:** 2015-06-28

**Authors:** Qi-Long Chen, Yi-Yu Lu, Jing-Hua Peng, Shu Dong, Bin Wei, Ya-Nan Song, Qian-Mei Zhou, Hui Zhang, Ping Liu, Shi-Bing Su

**Affiliations:** ^1^Research Center for TCM Complexity System, Shanghai University of TCM, Shanghai 201203, China; ^2^Institute of Liver Diseases, Shuguang Hospital Affiliated to Shanghai University of TCM, Shanghai 201203, China; ^3^Shanghai University of TCM, Shanghai 201203, China

## Abstract

Fuzheng-Huayu (FZHY) tablet was formulated based on Chinese medicine theory in treating liver fibrosis. A clinical trial has indicated that FZHY can against hepatitis B-caused liver cirrhosis (HBC), but the underlying mechanism of FZHY efficacy is unclear. Here, we report that miRNA expression levels are remarkably changed when FZHY formula was used in HBC patient's treatment as a paradigm of trials. Then, we functionally characterize the significant impact of potential kernel miRNAs by miRNA-target network analysis. Enrichment analysis show that the FZHY formula dramatically effecting the molecular regulated module in HBC. Thus, we infer that FZHY plays a critical function in HBC treatment process and directly regulated many important pathways, including but not limited to cell cycle, p53 signaling pathway, and TGF-*β* signaling pathway, suggesting a new strategy for investigating the molecular mechanism of FZHY treatment.

## 1. Introduction

Liver cirrhosis (LC) is a consequence of chronic liver disease characterized by liver fibrosis, scar tissue, and regenerative nodules, leading to the destruction of hepatic microstructure and liver dysfunction. Usually, cirrhosis is caused by hepatitis viruses, alcoholism, nonalcoholic steatohepatitis (NASH), and autoimmune liver disease as well as fatty liver disease. Noticeably, infection of hepatitis B virus (HBV) in human liver that induces the development of liver cirrhosis is increasing, annually, the HBV caused cirrhosis more than 1.5 million people in the world [1]. The five-year survival rate of patients with severe HBV caused cirrhosis only is about 50% [2] and rigorously and clinically lacks the effective drugs for the therapy of hepatitis B-caused cirrhosis in the past decades.

Fuzheng-Huayu (FZHY) tablet, a Chinese herbal formula, containing herbs such as* Radix Salvia miltiorrhiza*,* Cordyceps*,* Semen Persicae*, was formulated based on Chinese medicine theory in treating liver fibrosis and was approved. Pharmacological studies and clinical trials [3] have demonstrated that FZHY has a significant effect against liver fibrosis, in particular the effects observed from clinical trials in treating liver fibrosis caused by chronic hepatitis B. Furthermore, the actions on inhibition of hepatic stellate cell activation [4] and regulation of TGF-beta 1 signaling transduction pathway [5] also were effected for FZHY against liver fibrosis. In previous clinical trial, we have found the therapeutic efficacy of FZHY on hepatitis B-caused cirrhosis (HBC), but the underlying mechanism of FZHY efficacy is to a large extent still elusive.

MicroRNA (miRNA) is a class of small, endogenous, noncoding RNA molecules [6, 7], which suppress the translation of target mRNAs or induce mRNAs degradation [8–10]. Depending on the grade of concordance between miRNA sequence and target mRNA, the negative regulatory effect for target mRNAs more like as a rheostats to make fine-scale adjustments to protein output [11]. Generally, miRNAs have higher stability in circulation systems, tissue, and organ [12], and they are often detected in blood under pathological conditions caused by cell turnover, cell destruction, and pathological injury. Furthermore, miRNAs are relevance as regulators of gene expression, therefore affecting crucial processes in diseases development, especially, and offer great potential as biomarkers for diseases detection due to their remarkable stability in blood [12, 13].

In previous study, the multicenter, double blind, equal-randomized, and placebo-controlled trials were used to evaluate the curative effects of FZHY treatment in hepatitis B-caused cirrhosis (HBC). The results demonstrated that FZHY can clearly improve the TCM symptoms and quality of life of HBC patients. In this work, we hypothesized that miRNAs profiling in serum has potential as ideal biomarkers, which are associated with FZHY efficiency on HBC and thus focused on the miRNAs profiling analysis, the differences, and similarities in the FZHY and Placebo treatment in HBC. The aim was to investigate the molecular mechanism of FZHY efficacy on HBC through a regulation of miRNAs.

## 2. Materials and Methods

### 2.1. Clinical Specimens

The clinical serums of 6 HBC patients before trial and after trial (6 months) were collected, including 3 patients of FZHY intervention group and 3 patients of Placebo intervention. The selected criteria of samples including HA (hyaluronic acid), ALT (alanine aminotransferase), AST (aspartate aminotransferase), and Child-Pugh score in FZHY group were distinctly decreased compared to Placebo group. In addition, 7 serums of normal controls were randomly obtained from Shanghai Longhua Hospital. The diagnostic criteria of western medicine for HBC followed the guidelines that are defined by the Chinese Society of Hepatology and Chinese Society of Infectious Diseases in 2005 [14].

This research project was conducted according to the guidelines of the Declaration of Helsinki and the principles of Good Clinical Practice (China) and approved with the local ethics committee of Shanghai University of TCM. Furthermore, informed consent was received from all patients of this study. The clinical data of patients with CHB-caused cirrhosis were shown in Table 1.

### 2.2. Serum Sample Collection and RNA Isolation

All serum samples were from the peripheral venous blood of HBC subjects and healthy donors, which were immediately frozen in liquid nitrogen and then stored at −80°C. The RNAs in serum were extracted using a miRVana PARIS kit (Ambion, Austin, TX) according to the manufacturer's protocol and based on the RNase-free DNase I (Promega, Madison, WI) to eliminate DNA contamination. The concentration of RNAs isolated from serum ranged from 1.5 to 12 ng/*μ*L.

### 2.3. miRNA Microarray and Data Analysis

The miRNA profiles of 12 HBC subjects (6 before trial samples and 6 after trial samples) and 7 controls were generated using Agilent Human miRNA microarray V3 (Agilent Technologies Inc., USA). Hybridization signals were detected with the Agilent Microarray Scanner; the data were extracted using Feature Extraction V10.7 (Agilent Technologies, CA). All raw data were transformed to log2 and normalized each expression by zero mean and unit sample variance.

Using random variance model* t*-test of R package, the differential expression (DE) miRNAs were calculated among FZHY group, Placebo group, and Control group, where the fold-change >1.5 and *P* < 0.001 were considered to be significant. Heat map and hierarchical cluster analysis of expression data were performed using Cluster 3.0 and TreeView programs. Class prediction of samples was performed using a statistical algorithm of the support vector machine (SVM) incorporating differential expression (DE) miRNAs at a univariate parametric significance level of *P* = 0.01. The prediction rate was estimated via 10-fold and 10-time cross-validation and the bootstrap method for small sample data.

### 2.4. Identification of miRNA-Target Genes and miRNA-Target Network Constructing

The validated miRNA-target genes were predicted using three databases involving TarBase (v6.0) [15], miRecords (2013) [16], and miRTarBase (2013) [17], which hosted the largest collection of manually curate experimentally data. Furthermore, the programs of miRanda, miRDB, miRWalk, and RNAhybrid were used to predict the unverified miRNA-target genes, where *P* < 0.001 was considered to be significant. All predictions were merged and acted as final data for building miRNA-target network, of which the profiles were constructed using Cytoscape software (version 3.1). In the network, nodes represent miRNAs or target genes, and the edges represent the connection strength.

### 2.5. Enrichment Analysis of Target Genes

Of the inferred miRNA-target genes, those showing a significant (*P* < 0.05) expression difference among the samples before and after FZHY or Placebo treatment were analyzed for pathways involving these genes using DAVID online analysis [18, 19], and significance analysis was determined when *P* values were corrected for false discovery rate (FDR). Gene sets containing less than 5 genes overlapping were removed from the DAVID analysis. In our analysis, GO terms and pathways with an FDR-adjusted *P* value of less than 0.05 were retained.

## 3. Results

### 3.1. Differential Expressed miRNAs Regulated by FZHY Treatment in HBC

**Figure 1:**
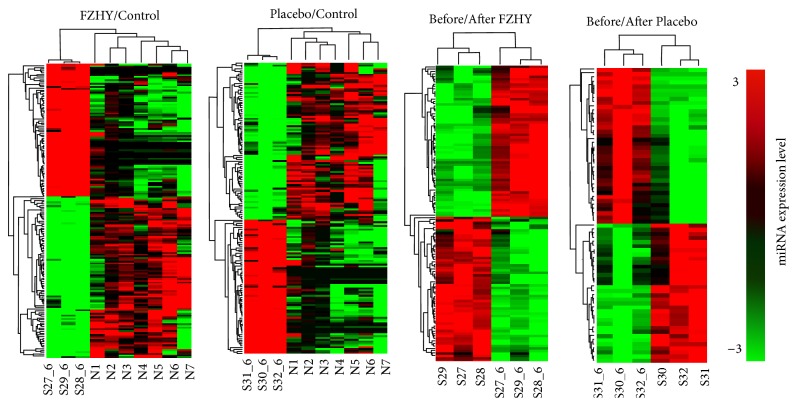
Heat map of differential expressed miRNAs among the FZHY/Control, Placebo/Control, Before/After FZHY treatment, and Before/After Placebo treatment was shown based on different colors, respectively. Red color represents miRNA upexpression and green color represents downexpression. Relationship among the samples was divided by binary tree classification and showed at the upper portion. Hierarchical cluster of miRNAs was displayed at nearside.

We calculated the miRNAs profiles among FZHY group, Placebo group, and Controls before trials. Before trials, there are 8 DE miRNAs between FZHY group and Controls and 9 DE miRNAs between Placebo group and Controls. However, after trials, 158 DE miRNAs were calculated between FZHY group and Controls (FZHY/Control), and 147 DE miRNAs were selected between Placebo group and Controls (Placebo/Control). Furthermore, 111 DE miRNAs were detected between before and after trials in FZHY group (Before/After FZHY), and 68 miRNAs were obtained in Placebo group (Before/After Placebo). The consecutive heat maps and hierarchical cluster showed the classification of miRNAs expression profiles in HBC compared to normal subjects (Figure 1).

Analysis of the DE miRNAs profiles showed that 138 miRNAs were overlapped between FZHY and Placebo groups after trials. Interestingly, 43 overlapped miRNAs were selected between the Before/After FZHY group and Placebo group. This result indicates that the overlapped miRNAs might play important regulated functions for FZHY treated process in HBC patients. To evaluate the variety of the overlapped miRNAs, we calculated the ratio value of each miRNA between FZHY and Placebo groups based on expression levels and obtained 58 miRNAs (ratio > 1). Similarly, 20 miRNAs (ratio > 1) were selected from Before/After FZHY and Placebo groups.

### 3.2. Overview of miRNA-Target Networks

**Figure 2:**
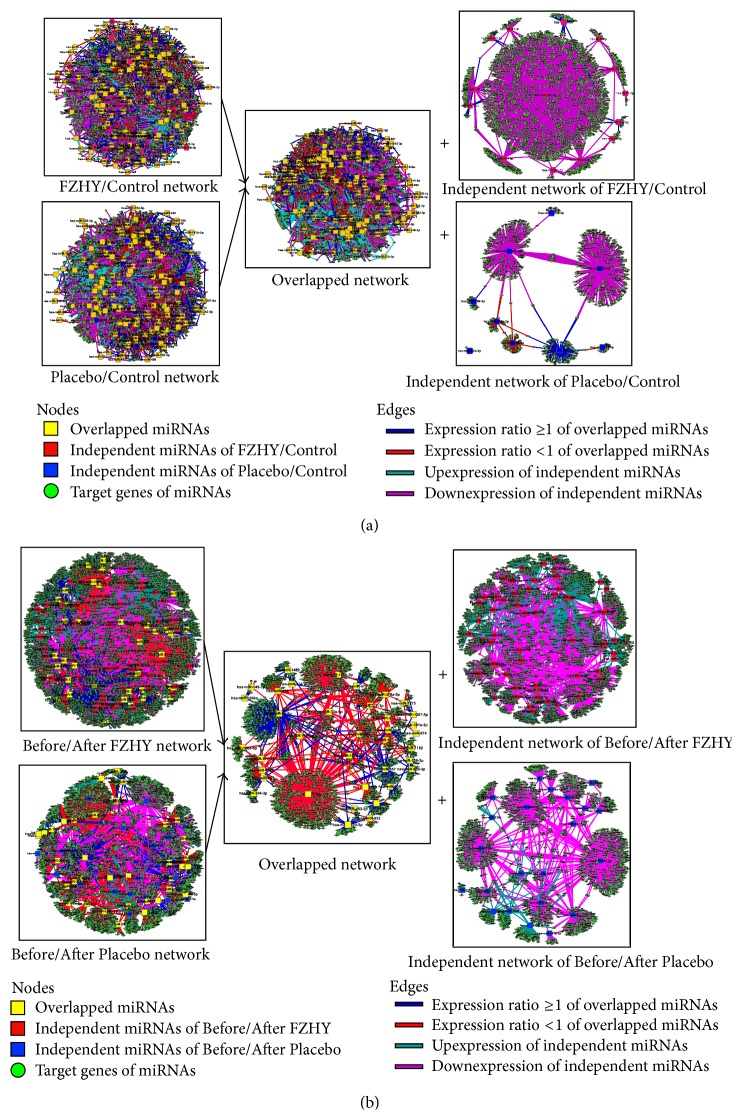
The global profiles of miRNA-target networks in FZHY and Placebo trials were showed. (a) The global profiles of FZHY/Control and Placebo/Control miRNA-target networks were constructed, respectively, and included the overlapped network and independent networks of FZHY/Control and Placebo/Control. (b) The global profiles of Before/After FZHY and Before/After Placebo miRNA-target networks were constructed, respectively, and included the overlapped network and independent networks of Before/After FZHY and Before/After Placebo.

The consecutive miRNA-target networks of each stage were constructed using the DE miRNAs and predicted target genes (Figure 2). To reveal the details of network, the global networks were divided into overlapped network and independent network in FZHY (FZHY/Control) and Placebo (Placebo/Control) groups (Figure 2(a)). Similarly, the Before/After FZHY and Before/After Placebo networks were also divided into the overlapped network and independent network, respectively (Figure 2(b)). By analysis of the nodes of network, we found that the independent networks only consist of 20 miRNAs (12.7%) and 9 miRNAs (6.1%) in FZHY (FZHY/Control) and Placebo (Placebo/Control) groups, respectively. However, the independent network consists of 68 miRNAs (61.3%) in Before/After FZHY group and 25 miRNAs (36.8%) in Before/After Placebo group. This phenomenon suggested that the diversity of miRNA expression levels has been changed between FZHY and Placebo groups.

Generally, miRNA inhibits translation or induces mRNA degradation by binding to the 3′-UTRs of target mRNAs [8]; here, we mainly focus on upexpression miRNAs for each stage. Following the network, we found that the topological profile of each network is more likely similar to “Medusa” model [20], which consists of regulatory core by hub nodes and is represented most prominently in the network. It indicates that the hub nodes of network are determinants of the realized gene expression profiles, but the periphery nodes that should be regulated are not regulated.

### 3.3. Potential Kernel miRNAs Selection

To estimate the hub nodes form networks, we have calculated the betweenness centrality (BC), closeness centrality (CC), and degree (*d*) of each node. Based on the value distribution, the thresholds of BC and CC were defined as 0.01 in the network, respectively. The candidate kernel nodes were selected by the conditions of BC ≥ 0.01, CC ≥ 0.01, and *d* ≥ 2 average (*d*). Thus, we detected 23 miRNAs in FZHY/Control, 19 miRNAs in Placebo/Control, 15 miRNAs in Before/After FZHY, and 8 miRNAs in Before/After Placebo groups. Interestingly, there are 19 overlapped miRNAs between FZHY/Control and Placebo/Control groups and 4 overlapped miRNAs between Before/After FZHY and Before/After Placebo groups. We calculated the overlapped miRNA expressed level ratio between FZHY/Control and Placebo/Control groups, as well as between Before/After FZHY and Before/After Placebo groups. To classify the overlapped miRNAs, the miRNAs expression ratio value were calculated, >1 represents this miRNA level was increased, whereas, ≤1 means it was decreased.

Then, these miRNAs were divided into two groups. Group A was associated with FZHY efficacy, which consists of the overlapped miRNAs (ratio < 1) and independent upexpression miRNAs of FZHY/Control and Before/After FZHY groups. Group B was associated with Placebo trial, including the overlapped miRNAs (ratio ≤ 1) and independent upexpression miRNAs of Placebo/Control and Before/After Placebo groups. Finally, 30 miRNAs were selected as potential kernel miRNA stringently associated with FZHY efficacy and 16 miRNAs highly correlated with Placebo trial (Table 2).

### 3.4. Enrichment Analysis for Potential Kernel miRNA Targets

**Figure 3:**
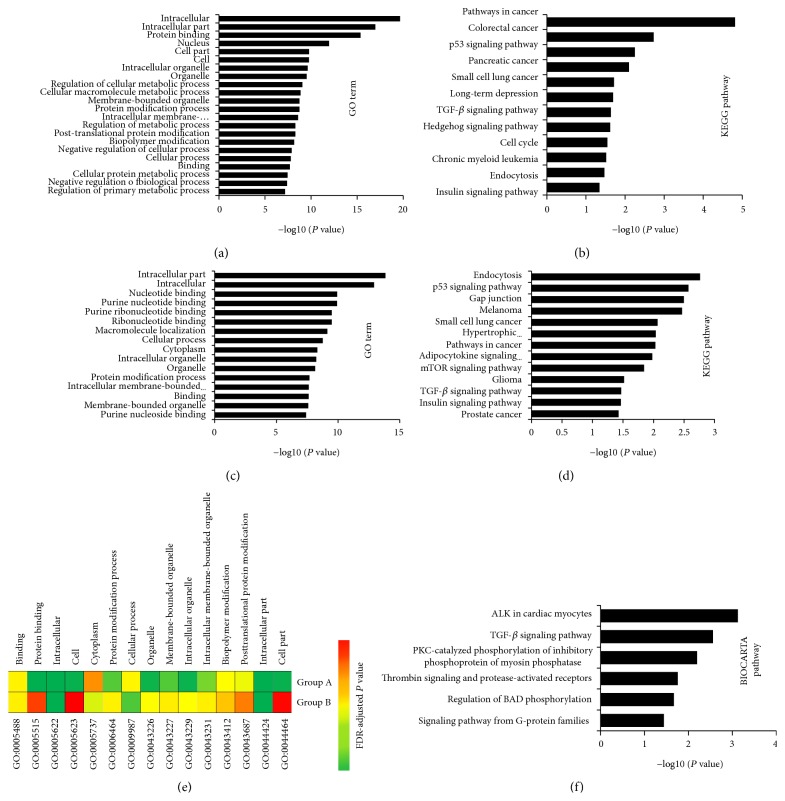
GO term and pathway by target genes of kernel miRNAs. Significant analysis was determined when *P* values were corrected for false discovery rate (FDR). Gene sets containing less than 5 genes overlapping were removed from the DAVID analysis. (a) Group A miRNA targets related GO term, (b) Group A miRNA targets related KEGG pathway, (c) Group B miRNA targets related GO term, (d) Group B miRNA targets related KEGG pathway, and (e) the distribution of overlapped GO terms between Group A and Group B (FDR-adjusted *P* value < 0.001) were showed. Colors were scaled according to the proportion of overlaps. (f) The BIOCARTA pathway of Group A miRNA targets, while Group B miRNA targets have not correlated with any pathway when *P* < 0.05.

To understand the potential kernel miRNAs holistically, we conducted functional enrichment analysis for the target genes of them using DAVID analysis [18, 19]. GO term (5% top terms) analysis reveals that Group A miRNA targets are mainly associated with intracellular, nucleus, protein binding, membrane-bounded organelle, protein modification process, posttranslational protein modification, cellular macromolecule metabolic process, cell, cellular process, regulation of cellular metabolic process, and negative regulation of cellular process (Figure 3(a)). Similarly, Group B miRNA targets are mainly associated with intracellular part, nucleotide binding, protein binding, purine nucleoside binding, ribonucleotide binding, intracellular membrane-bounded organelle, intracellular membrane-bounded organelle, macromolecule localization, cytoplasm, and cellular process (Figure 3(b)). Interestingly, there are 15 overlapped GO terms (*P* < 0.001) between Group A and Group B (Figure 3(e)).

Importantly, KEGG pathway analysis shows an impressive functional association of Group A miRNA targets with various cancer-related pathways, such as pathways in cancer (*P* = 2.09 × 10^−5^), colorectal cancer (*P* = 1.42 × 10^−3^), p53 signaling pathway (*P* = 4.55 × 10^−3^), small cell lung cancer (*P* = 1.58 × 10^−2^), endocytosis (*P* = 3.37 × 10^−2^), TGF-beta signaling pathway (*P* = 2.32 × 10^−2^), chronic myeloid leukemia (*P* = 2.58 × 10^−2^) as well as cell cycle (*P* = 2.31 × 10^−2^), and prostate cancer (*P* = 2.11 × 10^−2^) (Figure 3(c)). In Group B, except 6 overlapped pathways, the miRNA targets are also associated with gap junction (*P* = 3.18 × 10^−3^), melanoma (*P* = 3.41 × 10^−3^), hypertrophic cardiomyopathy (*P* = 9.17 × 10^−3^), adipocytokine signaling pathway (*P* = 1.05 × 10^−2^), mTOR signaling pathway (*P* = 1.43 × 10^−2^), prostate cancer (*P* = 3.73 × 10^−2^), and glioma (*P* = 3.04 × 10^−2^) (Figure 3(d)). Furthermore, BIOCARTA pathway only appeared in Group A (*P* < 0.05) and was mainly associated with ALK in cardiac myocytes (*P* = 7.57 × 10^−4^), TGF-*β* signaling pathway (*P* = 2.78 × 10^−3^), PKC-catalyzed phosphorylation (*P* = 6.39 × 10^−3^), thrombin signaling (*P* = 1.76 × 10^−2^), regulation of BAD phosphorylation (*P* = 2.15 × 10^−2^), and signaling pathway from G-protein families (*P* = 3.65 × 10^−2^) (Figure 3(f)).

**Figure 4:**
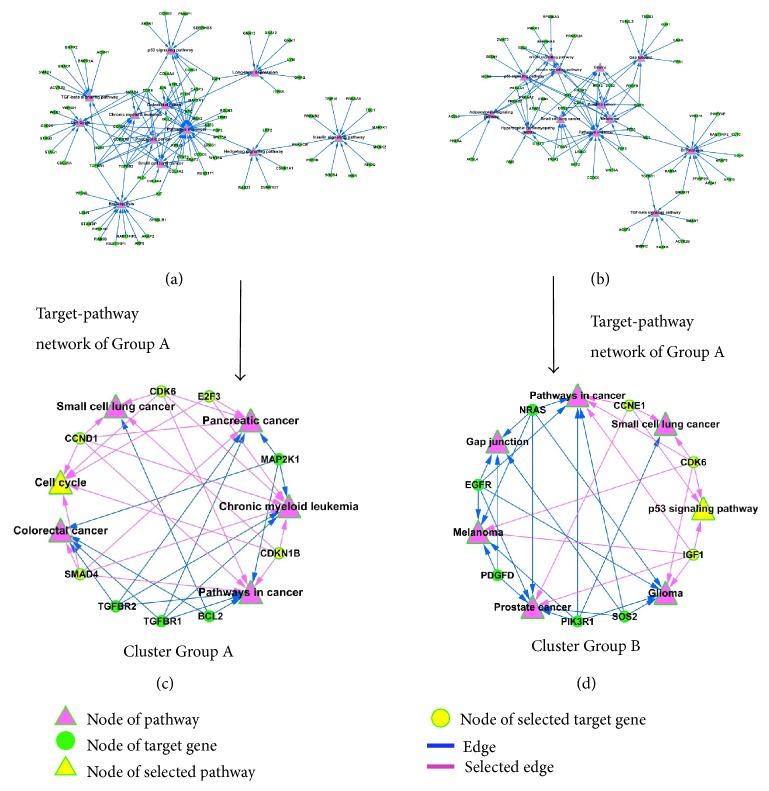
The global profile of target-pathway network and related cluster was showed. (a) Group A miRNA targets related target-pathway network, (b) Group B miRNA targets related target-pathway network, (c) the cluster of Group A miRNAs related target-pathway network, and (d) the cluster of Group B miRNAs related target-pathway network.

Subsequently, we constructed the target-pathway network using miRNA targets and related KEGG pathways (Figures 4(a) and 4(b)). The topological profiles of network were calculated using ClusterONE algorithm [21], which was defined as* P* value < 0.001, node size ≥ 5, and network density ≥ 0.05, and then obtained the kernel nodes related cluster from network. As showed in Figures 4(c) and 4(d), the cluster of Group A contained 6 pathways and 9 kernel genes, and the cluster of Group B contained 7 pathways and 8 kernel genes. Conspicuously, we notice that 5 genes (CDK6, E2F3, CCND1, SMAD4 and CDKN1B) are associated with cell cycle, colorectal cancer, pancreatic cancer, chronic myeloid leukemia, and small cell lung cancer in the cluster of Group A (Figure 4(c)). Interestingly, there are 3 genes (IGF1, CDK6, and CCNE1) which also acted in similar roles in the cluster of Group B, which was correlated with p53 signaling pathway, as well as glioma, prostate cancer, small cell lung cancer, pathways in cancer, and melanoma (Figure 4(d)). It suggested that the cell cycle might play an important role in FZHY treatment process, while p53 signaling pathway is a major component in Placebo trial.

## 4. Discussion

As a class of gene regulators, miRNA has an important combinatorial hallmark in gene regulation process; in particular, the exceptional stability of circulating miRNAs in serum is the basis of their value in clinical use [13]. In this work, we generated miRNAs expression maps of FZHY group and Placebo group by miRNA microarray and reported that the miRNAs levels were prominently changed in both of them. As shown in results, after 6-month treatment, the DE miRNAs are strongly increasing in FZHY (158 miRNAs) and Placebo groups (147 miRNAs). Although FZHY and Placebo groups have similar DE miRNAs, the ratio value of overlapped miRNAs suggested that there are great differences between FZHY and Placebo treatment and also indicated that these miRNAs are nonnegligible factors in the process of FZHY treatment. Subsequently, the consecutive miRNA-target networks were constructed, and the topological profiles are more likely similar to “Medusa” model; it suggests that the kernel nodes of network are determinants in the realized gene expression levels [22, 23].

Actually, the summation of gene expression and network connectivity can quantitative evaluated the module conservation in complex diseases [24], which provides a new avenue in understanding of molecular mechanism and distinguishing functional processes in disease progression [24, 25]. Here, we obtained 30 kernel miRNAs from network, which are highly correlated with the curative effects of FZHY in HBC treatment process (Table 2). FZHY is a Chinese herbal formula and contained many complex compounds, and we speculate that these kernel miRNAs might format a molecular group and holistically play important regulated functions in HBC treatment process. To understand the potential functions of these kernel miRNAs, we conducted enrichment analysis for the target genes of them. As shown in Figures 3(a) and 3(c), cellular macromolecule metabolic process, cellular process, and regulation of cellular metabolic process as well as negative regulation of cellular process are highly correlated with FZHY group; however, the Placebo group mainly associated with purine nucleoside binding, ribonucleotide binding, intracellular membrane-bounded organelle, intracellular membrane-bounded organelle, and macromolecule localization. Furthermore, the discrepant* P* values (PDR-adjusted) suggest that they might play different roles in the FZHY and Placebo trials process, although they have many overlapped GO terms (Figure 3(e)) and pathways. The results suggested that there were different molecular regulated modules between FZHY and Placebo treatment process in HBC.

To determine the biological consequence of pathway-mediated landscape in FZHY and Placebo trials, we constructed the target-pathway networks using kernel miRNA targets and KEGG pathways. The cluster results of network showed that cell cycle is an important pathway in FZHY treatment process, and p53 signaling pathway is a major component in Placebo treatment process. The imbalance of G1/S and G2/M phases of cell cycle is associated with dysfunction in hepatocarcinoma [26], while p53 signaling pathway is highly correlated with the pathogenesis of numerous cancer types [27]. It implicates that the FZHY can significantly increase the cancer-related miRNAs levels, then mediates the related target genes, and transforms the original progressions of cell cycle and p53 signaling pathway in HBC patients.

On the other hand, we noticed that the TGF-*β* signaling pathway commonly appeared in KEGG and BIOCARTA pathway of FZHY treatment group. TGF-*β* (transforming growth factor-beta) is an important regulatory tumor suppressor factor in epithelial cells [28]. TGF-*β*1 expression level was correlated with tumor progression, metastasis, angiogenesis, and poor prognostic outcome in various types of human cancer [29–32]. TGF-*β* also is a central regulator in chronic liver disease, which contributes to all stages of disease progression from initial liver injury through inflammation and fibrosis to cirrhosis and hepatocellular carcinoma [33]. We speculate that TGF-*β* signaling pathway might act as an important marker to discriminate the curative effects of FZHY and Placebo treatment in HBC patients and may contribute to fighting against liver cirrhosis.

## 5. Conclusion

In conclusion, FZHY formula can remarkably change miRNAs expression levels of HBC patients and mediate the related molecular regulated module in HBC treatment process. Here, we infer that FZHY plays a critical function in HBC treatment process and directly regulated many important pathways, including but not limited to cell cycle, p53 signaling pathway, and TGF-*β* signaling pathway. It provides us with a new clue to investigate the molecular mechanisms of FZHY treated HBC process.

## Figures and Tables

**Table 1:** Clinical data of patients with CHB-caused cirrhosis (HBC).

Patient ID	Age	Gender	Clinical types	Intervention types	HA before/after	ALT before/after	AST before/after	Child-Pugh score before/after
S27	56	M	HBC	FZHY	406/148.2	151/23	162/37	5/5
S28	57	M	HBC	FZHY	355.5/108.5	92/19	113/28	8/5
S29	59	F	HBC	FZHY	182.7/70.6	27/20	28/31	11/7
S30	73	M	HBC	Placebo	238.7/363.5	28/114	34/97	5/6
S31	47	F	HBC	Placebo	116/180.2	21/35	44/54	7/8
S32	51	M	HBC	Placebo	57.4/164.1	53/63	51/68	5/9

**Table 2:** The potential kernel miRNAs selected by FZHY and Placebo treatments.

Group A (FZHY related miRNAs)				Group B (Placebo related miRNAs)	
has-miR-1207-5p	has-miR-765	hsa-miR-19b-3p	hsa-miR-320d	hsa-miR-101-3p	hsa-miR-301b
has-miR-1268a	hsa-miR-101-3p	hsa-miR-21-5p	hsa-miR-340-5p	hsa-miR-1299	hsa-miR-335-5p
has-miR-134	hsa-miR-103a-3p	hsa-miR-221-3p	hsa-miR-374a-5p	hsa-miR-130a-3p	hsa-miR-33a-5p
has-miR-135a-3p	hsa-miR-128	hsa-miR-25-3p	hsa-miR-374b-5p	hsa-miR-142-3p	hsa-miR-340-5p
has-miR-1471	hsa-miR-142-5p	hsa-miR-26b-5p	hsa-miR-424-5p	hsa-miR-148b-3p	hsa-miR-363-3p
has-miR-26a-5p	hsa-miR-15a-5p	hsa-miR-27b-3p	hsa-miR-93-5p	hsa-miR-16-5p	hsa-miR-381-3p
has-miR-483-5p	hsa-miR-186-5p	hsa-miR-29c-3p		hsa-miR-19b-3p	hsa-miR-410
has-miR-663a	hsa-miR-19a-3p	hsa-miR-30e-3p		hsa-miR-21-5p	hsa-miR-497-5p
